# Association of the Triglyceride-Glucose Index With Established Cardiovascular Disease in Adults With Metabolic Dysfunction-Associated Steatotic Liver Disease: A Cross-Sectional Study

**DOI:** 10.7759/cureus.103062

**Published:** 2026-02-05

**Authors:** Soumayan Mondal, Sidharth S Pattnaik, Nihar Ranjan Mohanty, Sailendra Nayak, Ambika Mohanty, Shubhransu Patro

**Affiliations:** 1 Internal Medicine, Kalinga Institute of Medical Sciences, Bhubaneswar, IND; 2 Internal Medicine, SCB (Srirama Chandra Bhanja) Medical College and Hospital, Cuttack, IND

**Keywords:** cardiometabolic risk, cardiovascular disease, insulin resistance, metabolic dysfunction-associated steatotic liver disease (masld), nonalcoholic fatty liver disease (nafld), triglyceride-glucose index

## Abstract

Background

Metabolic dysfunction-associated steatotic liver disease (MASLD) is increasingly recognised as a multisystem disorder in which cardiovascular disease represents the leading cause of morbidity and mortality. The triglyceride-glucose (TyG) index is a simple surrogate marker of insulin resistance, but its association with established cardiovascular disease in individuals with MASLD has not been well characterised.

Methods

In this cross-sectional study, consecutive adults attending a general medicine outpatient clinic at a tertiary care teaching hospital between May 2023 and October 2025 underwent comprehensive metabolic, hepatic, and cardiovascular evaluation. MASLD was defined using contemporary consensus criteria. Established cardiovascular disease was defined a priori using objective diagnostic criteria, including electrocardiography, transthoracic echocardiography, and ankle-brachial index assessment. Multivariable logistic regression was performed to evaluate the association between the TyG index and established cardiovascular disease after adjustment for demographic and cardiometabolic risk factors. Model discrimination and dose-response relationships across TyG quartiles were assessed.

Results

Among 456 participants, 140 (30.7%) had established cardiovascular disease and 415 (91.0%) met criteria for MASLD. Each one-standard deviation increase in the TyG index was independently associated with established cardiovascular disease after multivariable adjustment, with an adjusted odds ratio of 3.05 (95% confidence interval 2.34-3.98). A graded increase in cardiovascular disease prevalence was observed across increasing TyG quartiles, demonstrating a strong dose-response relationship. The TyG-augmented model showed good discrimination, with an area under the receiver operating characteristic curve of 0.82.

Conclusions

In adults with MASLD, the TyG index is independently associated with established cardiovascular disease, demonstrates a clear dose-response relationship, and improves model discrimination beyond conventional risk factors. These findings support the role of the TyG index as a marker of cardiometabolic burden and highlight the importance of insulin resistance-focused cardiovascular risk assessment in MASLD.

## Introduction

Cardiovascular disease (CVD) remains the leading cause of morbidity and mortality worldwide and is tightly linked to metabolic disorders, including obesity, type 2 diabetes mellitus, dyslipidaemia, and hypertension [1]. These conditions frequently coexist and interact through shared pathophysiological mechanisms, resulting in a substantial and cumulative increase in cardiovascular risk.

The recent adoption of the term metabolic dysfunction-associated steatotic liver disease (MASLD) reflects a conceptual shift in fatty liver disease, emphasising its close association with systemic metabolic dysfunction rather than alcohol exposure alone [2]. MASLD is now recognised as the hepatic manifestation of insulin resistance and metabolic syndrome and affects a large proportion of adults globally [3]. Importantly, cardiovascular disease represents the leading cause of mortality in individuals with MASLD, exceeding liver-related complications in most cohorts [4,5].

Despite this strong epidemiological link, cardiovascular risk stratification in MASLD remains suboptimal. Traditional cardiovascular risk prediction models were largely derived from general populations and may not adequately capture the metabolic heterogeneity and insulin resistance burden within MASLD [6]. As a result, there is a need for simple, scalable markers that reflect the metabolic processes linking MASLD to clinically manifest cardiovascular disease.

Insulin resistance plays a central role in the pathogenesis of both MASLD and cardiovascular disease, promoting atherogenic dyslipidaemia, endothelial dysfunction, chronic low-grade inflammation, and myocardial structural changes [7]. Although direct measurement of insulin resistance using the hyperinsulinaemic-euglycaemic clamp is considered the reference standard, its complexity and cost preclude routine clinical application [8]. Consequently, surrogate indices derived from routinely available laboratory parameters have gained increasing clinical relevance.

The triglyceride-glucose (TyG) index, calculated from fasting triglyceride and glucose concentrations, has been validated as a reliable surrogate marker of insulin resistance and shows strong correlation with clamp-based measures [9]. By integrating abnormalities in both glucose and lipid metabolism, the TyG index reflects key pathophysiological processes underlying insulin resistance that are central to the development of MASLD and atherosclerotic cardiovascular disease. Prior studies have demonstrated associations between the TyG index and subclinical atherosclerosis, coronary artery disease, and adverse cardiovascular outcomes [10-12]. However, most existing data focus on surrogate cardiovascular markers or composite outcomes, are derived from selected populations, or do not incorporate contemporary MASLD definitions. Critically, the relationship between the TyG index and objectively defined, established cardiovascular disease in adults with MASLD has not been well characterised in real-world clinical settings.

Accordingly, the present study aimed to evaluate the association between the TyG index and established cardiovascular disease in adults attending a general medicine clinic, with a specific focus on individuals with MASLD. The primary objective was to determine whether the TyG index is independently associated with established cardiovascular disease after adjustment for traditional risk factors. Secondary objectives included assessment of dose-response relationships across TyG quartiles, evaluation of the incremental discriminatory value of TyG beyond conventional risk factors, and exploration of the association between TyG and subclinical cardiovascular abnormalities.

## Materials and methods

Study design, setting, and duration

This was a cross-sectional observational study conducted at a single tertiary care teaching hospital in India. The study population comprised consecutive adult patients attending the general medicine outpatient clinic, representing a non-screening, real-world clinical population rather than a health-check or surveillance cohort. Data were collected over a 24-month period from May 2023 to April 2025.

The study was approved by the institutional ethics committee, and all procedures were conducted in accordance with the Declaration of Helsinki. Written informed consent was obtained from all participants prior to enrolment.

Study population

Adults aged ≥18 years were eligible for inclusion if complete data were available for metabolic parameters, hepatic ultrasonography, and cardiovascular evaluation. Participants were excluded if they had chronic liver diseases other than steatotic liver disease, including viral hepatitis, autoimmune liver disease, or secondary causes of hepatic steatosis. Secondary causes of hepatic steatosis were excluded through detailed clinical history and medication review, including assessment for exposure to drugs known to cause hepatic steatosis (such as systemic corticosteroids, amiodarone, methotrexate, and tamoxifen), and by exclusion of alternative clinical or laboratory features suggestive of non-metabolic liver disease. Alcohol intake was assessed using a structured self-reported questionnaire, and individuals consuming >30 g/day of alcohol (men) or >20 g/day (women) were excluded in accordance with contemporary MASLD consensus recommendations [2].

Individuals with a prior history of coronary revascularisation (percutaneous coronary intervention or coronary artery bypass grafting) or stroke were excluded to avoid confounding from long-standing, treated cardiovascular disease. Participants without a prior cardiovascular diagnosis were retained to permit identification of previously unrecognised cardiovascular disease using uniform, objective evaluation at study entry.

Definition of MASLD

MASLD was defined according to international multisociety consensus criteria [2]. MASLD was diagnosed based on the presence of hepatic steatosis on ultrasonography, together with at least one metabolic risk factor.

Hepatic steatosis was identified using standard ultrasonographic criteria, including increased hepatic echogenicity relative to the renal cortex, attenuation of the ultrasound beam, and impaired visualisation of intrahepatic vessels. Ultrasonography was performed by a single experienced operator, blinded to clinical and cardiovascular data, using standardised techniques. Ultrasonography was used to identify the presence of hepatic steatosis; formal grading of steatosis severity was not incorporated into the analysis.

Metabolic risk factors included overweight or obesity (body mass index ≥25 kg/m² using Asian cut-offs), type 2 diabetes mellitus, hypertension, dyslipidaemia, or metabolic syndrome, consistent with MASLD consensus definitions [2]. Participants without ultrasonographic evidence of hepatic steatosis were classified as non-steatotic controls. MASLD was treated as a clinical exposure variable within a broader general medicine cohort, rather than as an inclusion criterion, allowing comparison with participants without hepatic steatosis.

Clinical and biochemical assessment

Baseline demographic characteristics, smoking status, and medical history were recorded at enrolment. Anthropometric measurements were obtained using standardised methods, and body mass index was calculated as weight in kilograms divided by height in metres squared. Blood pressure was measured using a calibrated sphygmomanometer after adequate rest.

Fasting blood samples were collected for measurement of plasma glucose, triglycerides, total cholesterol, low-density lipoprotein cholesterol, high-density lipoprotein cholesterol, and glycated haemoglobin, along with other routine biochemical parameters, using automated laboratory methods. All investigations, including laboratory tests, electrocardiography, echocardiography, and ankle-brachial index measurement, were performed at the time of study entry after eligibility criteria were fulfilled.

The TyG index was calculated using the established formula [9]: 

$\text{TyG index} = \mathrm{ln} (\text{fasting triglyceride (mg/dL)} \times \text{fasting plasma glucose (mg/dL)} / 2)$

For regression analyses, TyG was analysed as a standardised continuous variable (per standard deviation increase). For dose-response analyses, TyG values were categorised into quartiles based on the cohort distribution, using the 25th, 50th, and 75th percentile cut-off values.

Cardiovascular evaluation

All participants underwent structured cardiovascular evaluation, including resting 12-lead electrocardiography, transthoracic echocardiography, and ankle-brachial index measurement.

Electrocardiograms were recorded at rest and interpreted manually by a physician using predefined criteria [13]. Transthoracic echocardiography was performed by a single experienced operator following American Society of Echocardiography and European Society of Cardiology guidelines [14,15]. Left ventricular ejection fraction was assessed using standard methods. Ankle-brachial index was measured in both lower limbs using standardised techniques, and the lower value was used for analysis [16].

Outcome definitions

Primary Outcome: Established Cardiovascular Disease

The primary outcome was established cardiovascular disease, defined a priori as the presence of clinically manifest cardiovascular disease identified using objective diagnostic criteria at study entry. Established cardiovascular disease was considered present if participants had evidence of coronary heart disease, heart failure with reduced ejection fraction, peripheral artery disease, or atrial fibrillation. Coronary heart disease was defined by newly identified regional wall motion abnormalities on transthoracic echocardiography and/or new ischemic changes on resting electrocardiography, including pathological Q waves or ischemic ST-T changes, in individuals without a prior diagnosis of coronary artery disease, consistent with guideline-recognised criteria [13]. Heart failure with reduced ejection fraction was defined as a left ventricular ejection fraction of 40% or less, in accordance with contemporary heart failure guidelines [17,18]. Peripheral artery disease was defined by an ankle-brachial index of 0.90 or less in either lower limb [16]. Atrial fibrillation was identified on resting electrocardiography [18]. Isolated left ventricular hypertrophy, diastolic dysfunction, pulmonary hypertension, valvular heart disease, borderline ankle-brachial index values greater than 0.90 and less than 1.00, and heart failure with preserved or mildly reduced ejection fraction were not included in the primary outcome definition.

Secondary Outcome: Subclinical Cardiovascular Abnormalities

Subclinical cardiovascular abnormalities were defined among participants without established cardiovascular disease and included structural or functional cardiac changes not meeting criteria for overt disease.

These included left ventricular hypertrophy, defined according to guideline-recommended echocardiographic cut-offs for left ventricular mass index, and left ventricular diastolic dysfunction, defined using standard ASE/EACVI criteria incorporating mitral inflow parameters, tissue Doppler indices, and left atrial volume [14,15]. Borderline ankle-brachial index values (>0.90 to <1.00) were also included in this category.

Sample size and statistical analysis

The required sample size was estimated a priori using a prevalence-based approach. Based on population-level data reporting a prevalence of established cardiovascular disease of approximately 23% among adults with steatotic liver disease, the expected prevalence of established cardiovascular disease was set at 0.23 [5]. Assuming a two-sided α of 0.05, a Z value of 1.96, and an absolute precision of 4%, the minimum required sample size was calculated as 425 participants. Consecutive sampling was continued until the target sample size was exceeded.

Continuous variables are presented as mean ± standard deviation, and categorical variables as counts and percentages. Comparisons between groups were performed using the independent-samples t-test for continuous variables and the chi-square test for categorical variables.

Multivariable logistic regression analysis was conducted to evaluate the association between the TyG index and established cardiovascular disease. Covariates were prespecified based on biological plausibility and prior literature, including age, sex, diabetes mellitus, hypertension, smoking status, low-density lipoprotein cholesterol, body mass index, and MASLD status. The TyG index was added to this model to assess its independent association with the primary outcome. Adjusted odds ratios with 95% confidence intervals are reported.

Model discrimination was assessed using receiver operating characteristic analysis, and areas under the curve were compared between models with and without inclusion of TyG. Dose-response relationships across TyG quartiles were evaluated by trend analysis using Spearman's rank correlation.

Missing data were minimal; analyses were conducted using complete-case analysis. All analyses were performed using R version 4.3.2 (R Foundation for Statistical Computing, Vienna, Austria), and figures were generated using base R functions. A two-sided p-value <0.05 was considered statistically significant.

## Results

Study population

A total of 456 participants were included in the final analysis. Of these, 140 participants (30.7%) met criteria for established cardiovascular disease, while 316 participants (69.3%) had no evidence of established cardiovascular disease and served as the comparison group.

MASLD was present in 415 participants (91.0%) overall. All included participants had complete data available for metabolic parameters, hepatic ultrasonography, electrocardiography, echocardiography, and ankle-brachial index assessment.

Baseline characteristics

Baseline demographic, metabolic, and hepatic characteristics stratified by established cardiovascular disease status are presented in Table 1.

**Table 1:** Baseline clinical and metabolic characteristics stratified by established cardiovascular disease status Continuous variables are expressed as mean ± standard deviation and were compared using the independent-samples t-test. Categorical variables are expressed as counts and percentages and were compared using the chi-square test. Test statistics are reported as t values or chi-square (χ²) values, as appropriate. CVD, cardiovascular disease; LDL, low-density lipoprotein cholesterol; MASLD, metabolic dysfunction–associated steatotic liver disease; TyG, triglyceride–glucose index.

Variable	No established CVD (n = 316)	Established CVD (n = 140)	Test statistic	p-value
Age (years)	47.54 ± 14.62	46.61 ± 14.50	t = 0.63	0.527
Body mass index (kg/m²)	25.16 ± 4.97	25.82 ± 5.55	t = −1.21	0.206
LDL cholesterol (mg/dL)	119.17 ± 17.72	124.54 ± 18.77	t = −2.87	0.0036
TyG index	9.12 ± 0.51	9.72 ± 0.49	t = −11.91	<0.001
Male sex	204 (64.6%)	85 (60.7%)	χ² = 0.62	0.496
Diabetes mellitus	111 (35.1%)	69 (49.3%)	χ² = 8.14	0.0060
Hypertension	122 (38.6%)	79 (56.4%)	χ² = 12.50	<0.001
Smoking	52 (16.5%)	36 (25.7%)	χ² = 5.34	0.029
MASLD	278 (88.0%)	137 (97.9%)	χ² = 11.58	0.0013

Participants with established cardiovascular disease had a significantly higher mean TyG index compared with those without established cardiovascular disease (9.72 ± 0.49 vs 9.12 ± 0.51; p < 0.001). The prevalence of MASLD was also higher among participants with established cardiovascular disease (97.9% vs 88.0%; p = 0.001). Diabetes mellitus and hypertension were more common in the established cardiovascular disease group, whereas age and sex distribution did not differ significantly between groups.

Association between the TyG index and established cardiovascular disease

In multivariable logistic regression analysis adjusted for age, sex, diabetes mellitus, hypertension, smoking status, low-density lipoprotein cholesterol, body mass index, and MASLD status, the TyG index was independently associated with established cardiovascular disease (Table 2).

Each one standard deviation increase in the TyG index was associated with an adjusted odds ratio of 3.05 for established cardiovascular disease (95% CI 2.34-3.98; p = 1.75 × 10⁻¹⁶). Among the covariates included in the model, the TyG index demonstrated the largest adjusted odds ratio. MASLD status was not independently associated with established cardiovascular disease after multivariable adjustment.

Adjusted odds ratios and confidence intervals for all variables included in the model are shown in Table 2.

**Table 2:** Multivariable logistic regression analysis for established cardiovascular disease (n = 140) Adjusted odds ratios were derived from a multivariable logistic regression model including age, sex, diabetes mellitus, hypertension, smoking status, LDL cholesterol, body mass index, and MASLD status. The TyG index is expressed per one standard deviation increase. CI, confidence interval; LDL, low-density lipoprotein cholesterol; MASLD, metabolic dysfunction–associated steatotic liver disease; OR, odds ratio; TyG, triglyceride–glucose index.

Variable	Adjusted OR	95% CI	p-value
Age (per year)	0.99	0.98–1.01	0.525
Male sex	0.93	0.57–1.51	0.767
Diabetes mellitus	1.55	0.96–2.50	0.074
Hypertension	1.48	0.91–2.41	0.115
Smoking	1.06	0.60–1.86	0.854
LDL cholesterol	1.00	0.99–1.02	0.643
Body mass index	1.01	0.97–1.06	0.663
MASLD	1.88	0.51–6.98	0.347
TyG index (per SD increase)	3.05	2.34–3.98	<0.001

Model discrimination and performance

**Figure 1:**
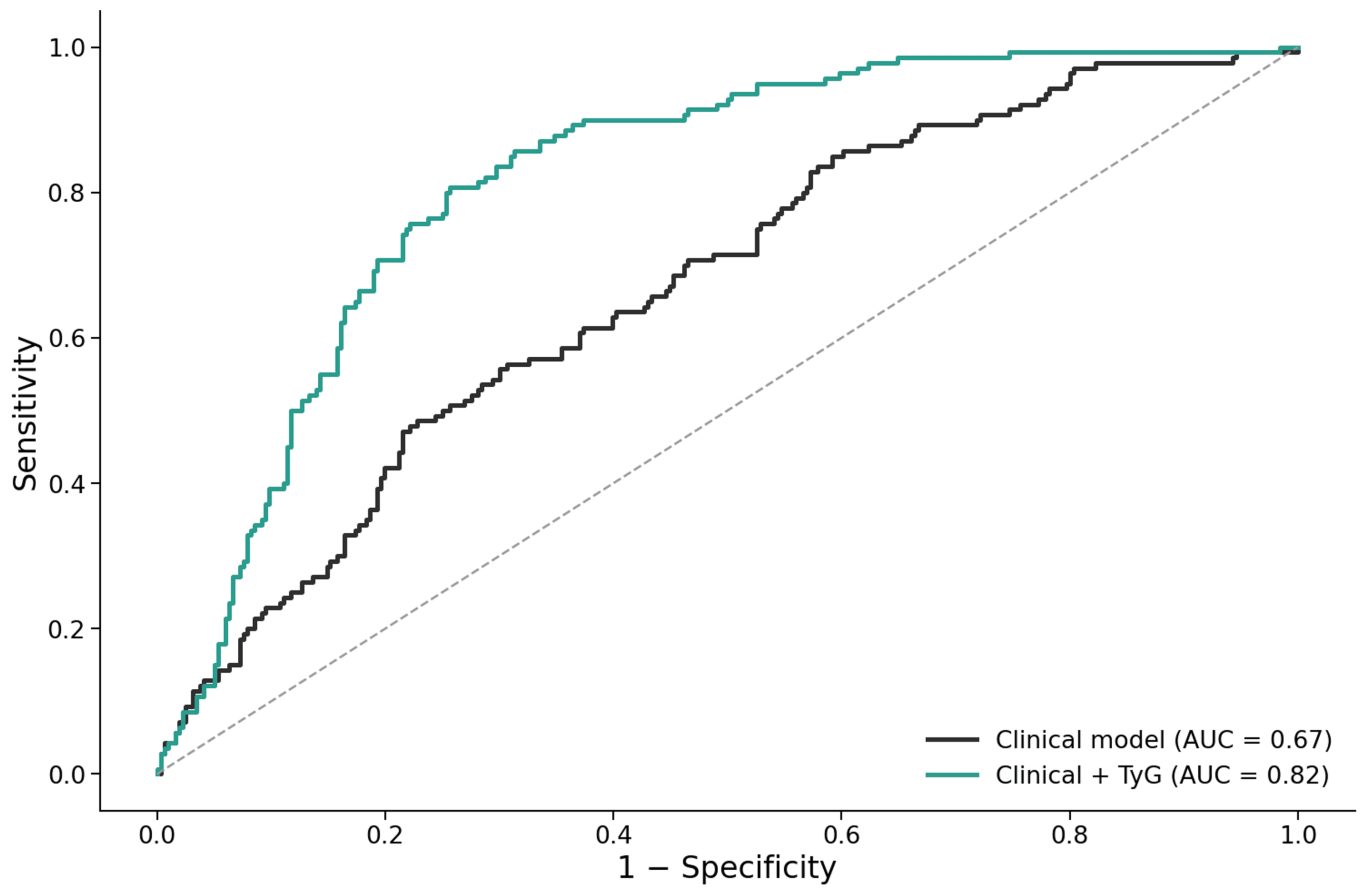
Receiver operating characteristic curves for prediction of established cardiovascular disease Receiver operating characteristic (ROC) curves comparing discrimination of a multivariable clinical risk model alone and the same model with addition of the triglyceride–glucose (TyG) index for prediction of established cardiovascular disease. The TyG-augmented model demonstrated improved discrimination, with an area under the ROC curve of 0.82. AUC, area under the curve; TyG, triglyceride–glucose index.

Receiver operating characteristic analysis demonstrated good discrimination for established cardiovascular disease (Figure 1). The clinical risk model showed moderate discriminative ability, which improved with the addition of the TyG index.

The TyG-augmented model achieved an area under the receiver operating characteristic curve of 0.82, with a Brier score of 0.16 and a McFadden pseudo-R² of 0.22, indicating good overall model performance (Appendices). Multicollinearity assessment showed higher variance inflation factors for variables reflecting metabolic burden, including body mass index, low-density lipoprotein cholesterol, and MASLD status, whereas the TyG index demonstrated minimal collinearity (Appendices).

Dose-response relationship across TyG quartiles

Participants were categorised into quartiles of the TyG index based on cohort distribution, with approximately equal numbers in each quartile. The prevalence of established cardiovascular disease increased progressively across increasing TyG quartiles (Table 3).

**Table 3:** Prevalence of established cardiovascular disease (hard CVD) across triglyceride–glucose index quartiles (total established CVD = 140) Quartiles were defined based on the cohort distribution of the triglyceride–glucose (TyG) index, with cut-off values corresponding to the 25th, 50th, and 75th percentiles (8.88, 9.18, and 9.74, respectively). Values are expressed as the number of participants with established hard cardiovascular disease and the corresponding percentage within each quartile. Trend analysis demonstrated a significant positive association across TyG quartiles (Spearman's r = 0.49; p < 0.001). CVD, cardiovascular disease; TyG, triglyceride–glucose index.

TyG quartile (exact cut-off range)	Total in quartile (n)	Established hard CVD, N (%)
Quartile 1 (TyG ≤ 8.88)	116	5 (4.3%)
Quartile 2 (TyG 8.89–9.18)	112	16 (14.3%)
Quartile 3 (TyG 9.19–9.74)	116	49 (42.2%)
Quartile 4 (TyG ≥ 9.75)	112	70 (62.5%)

The prevalence ranged from 4.3% in the lowest TyG quartile to 62.5% in the highest TyG quartile. Trend analysis demonstrated a strong positive association between TyG quartiles and established cardiovascular disease prevalence (Spearman's r = 0.49; p = 6.45 × 10⁻²⁹), consistent with a graded dose-response relationship across TyG quartiles.

Association between the TyG index and subclinical cardiovascular abnormalities

Among the 316 participants without established cardiovascular disease, the association between the TyG index and subclinical cardiovascular abnormalities was evaluated (Table 4).

**Table 4:** Multivariable logistic regression analysis for subclinical cardiovascular abnormalities among participants without established cardiovascular disease (n = 316) Multivariable logistic regression analysis examining the association between TyG index and subclinical cardiovascular abnormalities among participants without established cardiovascular disease. CI, confidence interval; LDL, low-density lipoprotein cholesterol; MASLD, metabolic dysfunction–associated steatotic liver disease; OR, odds ratio; TyG, triglyceride–glucose index.

Variable	Adjusted OR	95% CI	p-value
Age (per year)	1.01	0.99–1.03	0.338
Male sex	1.01	0.60–1.72	0.959
Diabetes mellitus	1.15	0.66–2.02	0.627
Hypertension	7.83	4.43–13.84	<0.001
Smoking	1.00	0.49–2.04	0.997
LDL cholesterol	1.01	0.99–1.02	0.362
Body mass index	1.07	1.01–1.13	0.021
MASLD	0.85	0.35–2.09	0.730
TyG index (per SD increase)	1.04	0.78–1.40	0.774

In multivariable analysis adjusted for the same clinical covariates, the TyG index was not independently associated with subclinical cardiovascular abnormalities. Hypertension and body mass index demonstrated significant associations with subclinical abnormalities, whereas MASLD status did not.

Summary of results

In this cohort of adults attending a general medicine outpatient clinic, the TyG index was independently associated with established cardiovascular disease, demonstrated a marked dose-response relationship across quartiles, and improved model discrimination when added to conventional clinical risk factors. Associations between TyG and subclinical cardiovascular abnormalities were weaker and not statistically significant after adjustment.

## Discussion

In this cross-sectional study of adults attending a general medicine outpatient clinic, the TyG index demonstrated a strong and independent association with established cardiovascular disease in individuals with MASLD. Each one-standard deviation increase in TyG was associated with approximately threefold higher odds of established cardiovascular disease after adjustment for age, sex, diabetes mellitus, hypertension, smoking status, lipid parameters, body mass index, and MASLD status itself. The association was supported by a clear dose-response relationship across TyG quartiles and by good overall model performance, including an area under the receiver operating characteristic curve of 0.82. Together, these findings suggest that TyG captures a clinically relevant dimension of cardiometabolic burden that is not fully reflected by conventional risk factors or by hepatic steatosis alone.

TyG as a marker of cardiometabolic burden in MASLD

MASLD is increasingly recognised as a multisystem condition in which cardiovascular disease represents the dominant determinant of long-term outcomes rather than liver-related complications [4,5]. However, MASLD encompasses a heterogeneous population with wide variability in metabolic risk. In the present study, MASLD status was associated with established cardiovascular disease on univariable analysis but lost statistical significance after multivariable adjustment, whereas the TyG index retained a strong and independent association. This finding suggests that insulin resistance burden, rather than hepatic steatosis per se, may be a more proximate driver of cardiovascular disease within the MASLD spectrum.

This interpretation is biologically plausible. Insulin resistance promotes atherogenic dyslipidaemia, endothelial dysfunction, vascular inflammation, and adverse myocardial remodelling, all of which contribute directly to cardiovascular disease pathogenesis [7]. At a molecular level, insulin resistance is associated with increased lipotoxicity, oxidative stress, and pro-inflammatory signalling, which link hepatic metabolic dysfunction to vascular and myocardial injury. Hepatic steatosis may therefore represent a marker of metabolic dysfunction, while TyG provides a more quantitative reflection of the underlying insulin-resistant state that drives cardiovascular injury. Similar dissociations between liver fat content and cardiovascular risk have been reported in prior studies, which have shown stronger associations for insulin resistance measures than for steatosis alone [19,20].

Comparison with existing TyG literature

Previous studies have reported associations between the TyG index and a range of cardiovascular outcomes, including coronary artery disease, arterial stiffness, and incident cardiovascular events [10-12]. However, most prior investigations focused on surrogate markers, composite endpoints, or selected cardiology cohorts, and few incorporated contemporary definitions of metabolic liver disease. Importantly, many studies did not distinguish between subclinical and overt cardiovascular disease or relied on self-reported outcomes. Accordingly, the TyG index should be interpreted as a marker of cardiometabolic risk rather than as a standalone diagnostic tool for cardiovascular disease.

The present study extends existing evidence by demonstrating a robust association between TyG and objectively defined, established cardiovascular disease using multimodal assessment, including electrocardiography, echocardiography, and ankle-brachial index measurement. In addition, the use of MASLD terminology situates these findings within the current conceptual framework of metabolic liver disease, allowing more precise interpretation of cardiometabolic risk in this population.

Dose-response relationship and biological plausibility

The progressive increase in the prevalence of established cardiovascular disease across TyG quartiles, from approximately 4% in the lowest quartile to over 60% in the highest quartile, provides further support for a biologically meaningful relationship. The strong trend statistic observed reinforces that the association is not driven by extreme values or modelling artefacts. Such graded relationships between TyG and cardiovascular risk have also been described in population-based cohorts and studies of coronary artery disease severity [10,11], supporting the consistency of this association across settings.

Model performance and statistical robustness

Beyond statistical significance, the TyG-augmented model demonstrated good overall performance, with an area under the receiver operating characteristic curve of 0.82 and acceptable calibration as reflected by the Brier score and McFadden pseudo-R². These values are comparable to, or better than, many established clinical risk models applied in cross-sectional cardiometabolic research.

Multicollinearity assessment showed higher variance inflation factors for variables reflecting overlapping aspects of metabolic dysfunction, including body mass index, low-density lipoprotein cholesterol, and MASLD status. Importantly, the TyG index demonstrated minimal collinearity, indicating that it captures information distinct from these traditional measures. This finding strengthens the interpretation that TyG functions as an integrative marker of cardiometabolic burden rather than a redundant proxy for existing risk factors.

Subclinical cardiovascular abnormalities

In contrast to its strong association with established cardiovascular disease, the TyG index was not independently associated with subclinical cardiovascular abnormalities after multivariable adjustment. Several explanations are possible. Subclinical cardiovascular phenotypes, such as diastolic dysfunction or left ventricular hypertrophy, are heterogeneous and may reflect diverse pathophysiological processes not uniformly driven by insulin resistance. In addition, cross-sectional assessment may have limited sensitivity to detect early disease stages. The stronger association observed for overt cardiovascular disease suggests that TyG may be more closely linked to advanced cardiometabolic injury rather than to early structural changes.

South Asian context and global relevance

The present findings are particularly relevant in the South Asian context. Individuals from South Asian populations exhibit a higher burden of insulin resistance and cardiometabolic risk at lower body mass index thresholds compared with Western populations [21]. MASLD is highly prevalent in this region, and cardiovascular disease often presents at a younger age. In such settings, markers that reflect insulin resistance rather than adiposity alone may be especially informative. While this study was conducted in an Indian cohort, the underlying mechanisms linking TyG to cardiovascular disease are global in nature, supporting broader applicability of these findings.

Clinical implications

The TyG index is inexpensive, widely available, and reproducible, requiring only fasting glucose and triglyceride measurements. Within the context of MASLD, TyG may serve as a marker of cardiometabolic burden that helps identify individuals at higher cardiovascular risk who may benefit from closer cardiovascular evaluation and aggressive risk factor modification. Importantly, TyG should not be viewed as a diagnostic or screening tool for cardiovascular disease, but rather as an adjunct to clinical assessment that enriches risk stratification in metabolically vulnerable populations.

Strengths and limitations

Key strengths of this study include the use of contemporary MASLD definitions, objective and multimodal assessment of established cardiovascular disease, prespecified multivariable modelling, and comprehensive evaluation of model performance. The inclusion of a real-world, non-screening general medicine population enhances clinical relevance.

Several limitations should be acknowledged. The cross-sectional design precludes assessment of temporality and causal inference. The composite definition of established cardiovascular disease includes heterogeneous phenotypes, which may differ in their relationship with insulin resistance. The study was conducted at a single tertiary care centre, potentially limiting generalisability. The absence of a population-based control group and the outpatient clinic-based sampling may introduce selection bias, limiting external validity. Electrocardiographic ischaemic changes were identified on resting ECG and may not capture all forms of coronary disease. Residual confounding cannot be excluded, and direct measurement of insulin resistance was not performed. Liver enzymes and fibrosis indices were not incorporated into the analysis, as the study's focus was on cardiometabolic risk rather than liver disease staging.

Future directions

Prospective studies are needed to evaluate whether TyG predicts incident cardiovascular events and to assess the utility of longitudinal changes in TyG for risk stratification. Integration of TyG into existing cardiovascular risk models and evaluation of its incremental clinical utility in diverse populations warrant further investigation.

## Conclusions

In adults with MASLD, the TyG index is independently associated with established cardiovascular disease, demonstrates a strong dose-response relationship, and improves model discrimination beyond conventional risk factors. These findings support the role of TyG as a marker of cardiometabolic burden and highlight the importance of insulin resistance-focused risk assessment in MASLD.

## Appendices

**Table 5:** Performance metrics of the multivariable logistic regression model for prediction of established cardiovascular disease Model performance was assessed for the TyG-augmented multivariable logistic regression model predicting established cardiovascular disease. Discrimination was evaluated using the area under the receiver operating characteristic curve (AUC). Overall model accuracy and calibration were assessed using the Brier score. Global model fit was evaluated using McFadden pseudo-R². AUC, area under the curve; TyG, triglyceride–glucose.

Metric	Value
Area under the receiver operating characteristic curve (AUC)	0.82
Brier score	0.16
McFadden pseudo-R²	0.22

**Table 6:** Multicollinearity diagnostics (variance inflation factors) for covariates included in the multivariable logistic regression model Multicollinearity among covariates included in the multivariable logistic regression model for established cardiovascular disease was assessed using variance inflation factors (VIFs). Higher VIF values were observed for variables reflecting overlapping components of metabolic dysfunction. The triglyceride–glucose (TyG) index demonstrated minimal collinearity, indicating that it provides information largely independent of conventional metabolic risk factors. The model was adjusted for age, sex, diabetes mellitus, hypertension, smoking status, low-density lipoprotein cholesterol, body mass index, MASLD status, and the TyG index. MASLD, metabolic dysfunction–associated steatotic liver disease; SD, standard deviation; TyG, triglyceride–glucose; VIF, variance inflation factor.

Variable	Variance inflation factor (VIF)
Age	9.54
Sex (male)	2.69
Diabetes mellitus	1.80
Hypertension	2.06
Smoking	1.31
Low-density lipoprotein cholesterol	24.23
Body mass index	21.65
Metabolic dysfunction-associated steatotic liver disease	13.81
Triglyceride–glucose index (per SD increase)	1.09
